# Radiosensitization in prostate cancer: mechanisms and targets

**DOI:** 10.1186/1471-2490-13-4

**Published:** 2013-01-26

**Authors:** Diego A Palacios, Makito Miyake, Charles J Rosser

**Affiliations:** 1 Section of Urologic Oncology, MD Anderson Cancer Center Orlando, Orlando, FL 32806, USA; 2 Cancer Research Institute, MD Anderson Cancer Center Orlando, Orlando, FL 32827, USA

**Keywords:** Cancer, Prostate, Radiation, Radiosensitizer

## Abstract

Prostate cancer is the second most commonly diagnosed cancer in American men over the age of 45 years and is the third most common cause of cancer related deaths in American men. In 2012 it is estimated that 241,740 men will be diagnosed with prostate cancer and 28,170 men will succumb to prostate cancer. Currently, radiation therapy is one of the most common definitive treatment options for localized prostate cancer. However, significant number of patients undergoing radiation therapy will develop locally persistent/recurrent tumours. The varying response rates to radiation may be due to 1) tumor microenvironment, 2) tumor stage/grade, 3) modality used to deliver radiation, and 4) dose of radiation. Higher doses of radiation has not always proved to be effective and have been associated with increased morbidity. Compounds designed to enhance the killing effects of radiation, radiosensitizers, have been extensively investigated over the past decade. The development of radiosensitizing agents could improve survival, improve quality of life and reduce costs, thus benefiting both patients and healthcare systems. Herin, we shall review the role and mechanisms of various agents that can sensitize tumours, specifically prostate cancer.

## Review

### Introduction

In 2012 it is estimated that 241,740 men will be diagnosed with prostate cancer and 28,170 men will succumb to prostate cancer [1]. The lifetime risk of being diagnosed with prostate cancer is 1 in 6 [2]. Prostate cancer is the second most commonly diagnosed cancer in American men over the age of 45 years and is the third most common cause of cancer related deaths in American men [1,3]. The majority of men with newly diagnosed localized prostate cancer may be eligible for active surveillance, surgery (prostatectomy or cryoablation), or radiation therapy (external beam or brachytherapy) either alone or in combination with androgen deprivation therapy. This review will focus on radiation therapy, which is a common treatment option for localized prostate cancer. Radiation therapies, external beam, proton beam, and interstitial brachytherapy, are becoming increasingly in demand by patients [4,5] as a means to avoid major surgery and the side effects and convalescence associated with surgery [6].

Radiation therapy in prostate cancer is by no means innocuous and is associated with urinary and bowel side effects. Furthermore, it has been reported that based on the clinical presentation of the cancer, only 33-66% of patients undergoing radiation therapy are disease-free five years after the initial procedure, this includes more contemporary series addressing dose escalation (*i*.*e*., ≥78 Gy) [7-12]. The varying response rates to radiation may be due to 1) modality used to deliver radiation, 2) dose of radiation, 3) tumor stage/grade, 4) confounding medical co-morbidities and 4) intrinsic microenvironment of the tumor. Herein, we will discuss the intrinsic microenvironment of tumors and how radiosensitizing agents (*i*.*e*., drugs that can enhance the effectiveness of radiation, kill to the target tissue) may be used to improve the efficacy of radiation therapy [13].

### Cellular effects of ionizing radiation

To understand the mechanism and role of various radiosensitizing agents, it is important to review briefly the cellular response to radiation. Radiation is clinically administered either by an external source, linear accelerator, directed toward the tumor or an internal source, radioactive decay from within the tumor [13]. Five mechanisms have been described to explain the way radiation interacts with matter, including coherent scattering, the photoelectric effect, the Compton effect, the pair production, and the photodisintegration [14]. However, the Compton effect is widely viewed as the mode of interaction most relevant for the range of energies used in clinical radiation therapy. In the Compton effect, the observed biologic effect results from photons creating multiple ionizations by ejection of electrons from the target biomolecule [15]. In this regard, the extent of biologic effects in cells after exposure to ionized radiation is largely due to oxygen with the subsequent production of free radicals. These free radicals can break chemical bonds present in critical cell structures and molecules, namely cellular DNA.

**Figure 1:**
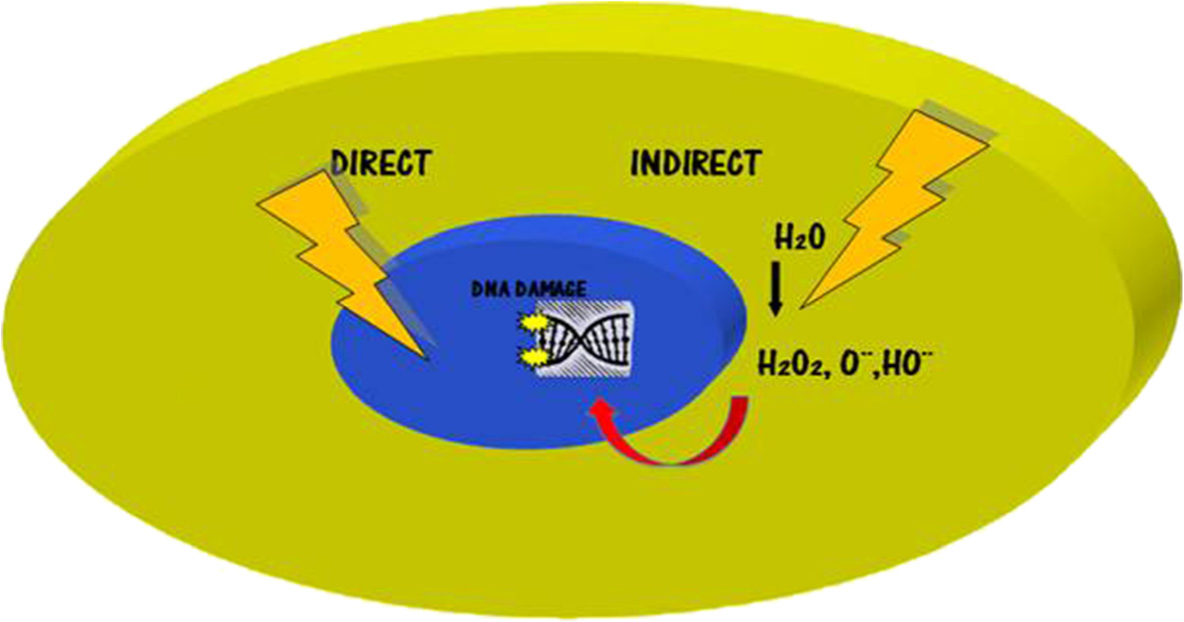
**Cellular Effects of Ionizing Radiation.** Photons of radiation produce direct DNA damage, but it is less likely than indirect damage, where photons eject electrons from target biomolecules in the cytoplasm, creating multiple ionizations, especially in oxygen compounds and producing free radicals, which break DNA.

DNA is by far the critical target for the biologic effects of radiation. Cell death is strongly associated with the extent of DNA damage [16,17] with radiosensitivity being more pronounced in cells that cannot effectively repair DNA damage [18-20]. Furthermore, cell death occurs at a higher rate when radiation is focused on the nucleus as opposed to the cytoplasm [21-23]. Disruption of DNA takes place via both direct and indirect effect of radiation. Taking into account the size of the cell and the small amount of DNA in the cell, most of the damage occurs indirectly [24]. This means that the photons of radiation are less likely to directly damage the DNA, but more likely to ionize surrounding molecules, which subsequently destabilize nucleic acids [25]. The mechanism behind oxygen radiosensitizing is related to this process of indirect damage (Figure 1). The presence of oxygen in irradiated tissues prevents repair of free radical-induced damage by forming irreversible peroxides in the fractured molecules. This process is sometimes called “fixing” the radiation [26]. Many types of damage to DNA molecules, dimers, adducts, oxidative base damage, intra- and intercross-links, DNA-protein cross-links, single- and double-strand breaks can be detected after radiation exposure.

According to the Compton effect, multiple ionizations are created and this leads to multiple free radical formation [13,15]. It is believed that these radicals are produced in clusters and in discrete areas. Thus, it is thought that the multiple broken bonds and subsequent nucleic acids damage are likely to be clustered as well. Often, the term “locally multiply damaged site” is used to refer to this phenomenon [27]. It has been suggested that clustered DNA damage is critical to clinically significant effects [27,28]. These effects truly depend on the cellular response following a radiation insult, including the ability to repair DNA damage and to activate survival mechanisms.

### Cellular detection of DNA damage

**Figure 2:**
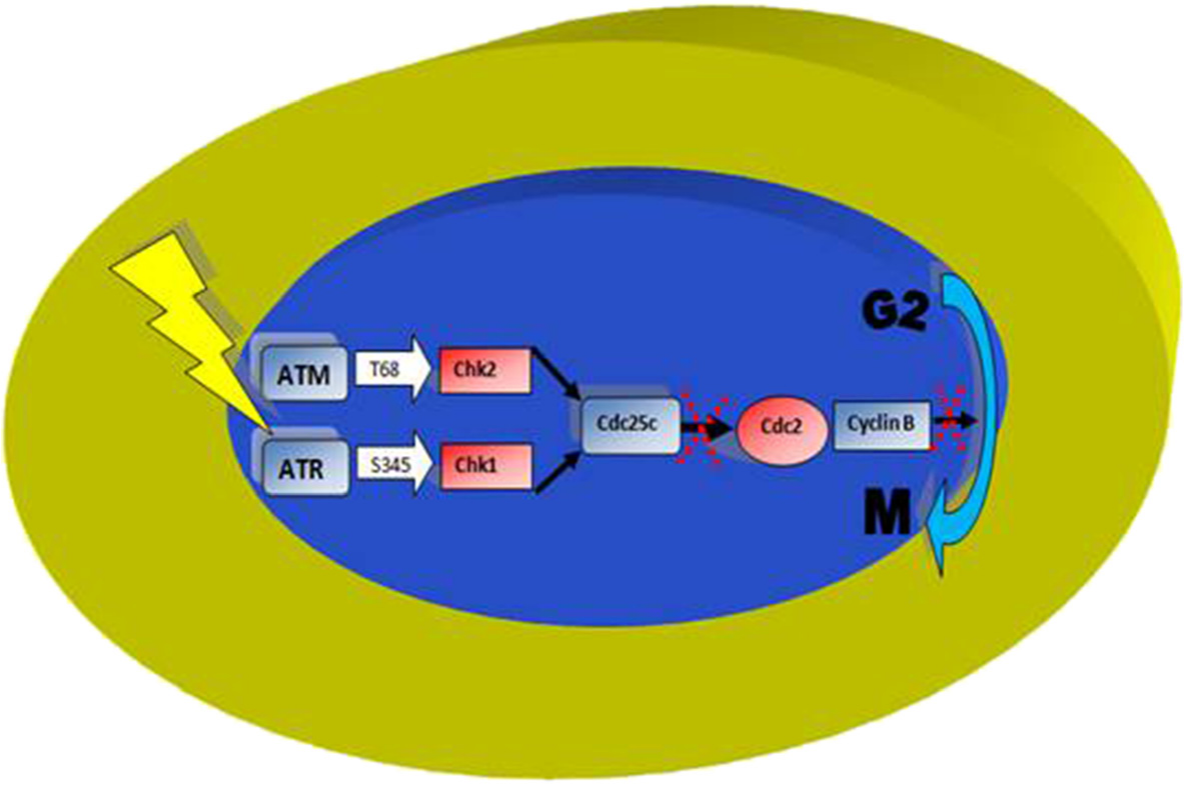
**G2**/**M Arrest after Ionizing Radiation DNA Damage.** Activation of ATM and ATR by DNA damage produces G2 arrest. Once DNA is repaired Cdc25c is inactivated which stimulates Cdc2 and enhances cell entry into mitosis by Cyclin B.

Shortly after exposure to ionizing radiation, a signal is transmitted to the regulators of the cell cycle machinery and the sensors of DNA damage. Cells with damaged DNA undergo G2/M cell cycle arrest (Figure 2). During this cell cycle arrest, the cells can either 1) repair and proceed through the cell cycle, 2) not repair and stay arrested, or 3) not repair and undergo apoptosis [29]. Cells under hypoxic conditions (*i*.*e*., unable to generate free radicals) show much less sensitivity to radiation as opposed to well oxygenated cells [30]. It then becomes intuitive that radiation effects are directly related with blood flow and oxygen concentration of the target tissue [31].

Several mechanisms have been proposed for the cascade leading to the cellular recognition of a radiation insult. Rad17-RFC, 9-1-1, and MRN complexes are three families of proteins that have been implicated in the initial sensing of DNA damage [32-35]. The Rad17-RFC complex is composed of the protein Rad17 and four subunits of replication factor C (Rfc2, Rfc3, Rfc4, Rfc5) [33]. The 9-1-1 complex consists of proteins Rad9, Rad1, and Hus1 [34]. This complex is thought to be more involved in single-strand DNA damage [36]. The MRN complex consists of proteins Mre11, Rad50, and Nbs1, and it is thought to be more specific to double-strand DNA damage and homologous repair [35,37]. These complexes are able to dock on fractured DNA or near the site of damaged DNA and transmit signals downstream to the transducers, which are members of the phosphoinositide 3-kinaserelated kinase such as ATM (ataxia-telangiectesia mutated protein) and ATR [32].

While ATM and ATR have some overlapping activities, they are activated by separate signals and by different types of DNA damage [38,39]. In this regard, ATM is more the central orchestrator of the cascade. In addition, some evidence implicates ATM/ATR as being involved in the initial sensing of DNA damage [40,41]. When ATM and ATR are activated, they relay the signal to various downstream effectors that mediate cell cycle arrest, DNA repair, and apoptosis. The specific effectors will be discussed according to the cellular response.

### Cell cycle arrest

Progression through the cell cycle is carefully coordinated by a series of events that culminate in DNA synthesis and cellular division. The control of multiplication relies on accelerating and braking mechanisms, which act on driving the cell cycle leading to mitosis. There are many checkpoints throughout the cell cycle that can prevent important cycle transitions until the integrity of the DNA is ensured [42-45]. Cells respond to ionizing radiation is variable, depending on when the cell is exposed to radiation in its cell cycle. Cells are extremely sensitive to radiation during mitosis in which there is no DNA repair [46-49].

DNA damage can activate multiple pathways that eventually lead to G1 arrest. When ATM is activated, it transduces signals to some key effector molecules. ATM stabilizes p53 by phosphorylating its serine-15, whereas it also adds a phosphate group on serine 395 of MDM2 [50]. In this context, phosphorylation of MDM2 prevents p53-MDM2 nuclear export and degradation of p53 [32,50]. ATM is also known to phosphorylate Chk2, which subsequently phosphorylates p53 on serine 20 [51]. This extra phosphorylation further prevents interaction of p53 and MDM2. At this point, the net result is more available nuclear p53, which is free to activate p21, a major inhibitor of the cyclin E-CDK2 complex [52]. This inhibited complex is important for G1 transition because it normally phosphorylates pRb and leads to the subsequent release of E2F [53,54]. ATM is also the coordinator of a p53/p21 independent G1 arrest pathway. When ATM activates Chk2, this kinase phosphorylates cdc25A, which is primed for ubiquination and subsequent degradation [55]. This is significant because cdc25A is a phosphatase that removes inhibitory phosphates from CDK2 and CDK4, which both are important for G1 phase progression molecules [32]. During the G1 phase, irradiated cells have been shown to be radioresistant, but their radiosensitivity increases at the end of this phase [56,57].

The most radioresistant phase of the cell cycle is the S phase, when cellular DNA is replicating [58,59]. During this phase, numerous nuclear machineries are available to repair and confirm the integrity of the DNA. However, if double-strand DNA damage occurs it can retard the replication of DNA and thus halt further DNA synthesis. ATM is again the key protein involved in this checkpoint [60,61]. The main pathway involves facilitating the degradation of the phosphatase cdc25A. This phosphatase activity is crucial for the function of cyclin E/A-CDK2 complexes, which oversee progression through the S-phase.

Finally and most commonly, irradiated cells can be blocked in the G2/M phase, which post mitosis is the next most sensitive phase in the cell cycle. Multiple pathways are involved for this arrest, and the complete details are not yet known [32]. However, the final step in this pathway is deactivation of the cyclin B-CDK1 complex, which orchestrates the G2/M transition [62,63]. As with the pathways discussed above, activation or deactivation of the CDK1 complex is determined by the specific site of phosphorylation. The ATM/CDC25A pathway is also important here because cdc25A is an activator of cyclin B-CDK1 complex [64]. Through p53, ATM activates p21, which is an inhibitor of an activator of CDK1, namely CAK (CDK activating kinase) [61].

### DNA repair

As described above, irradiated cells sense DNA damage, which eventually activates the mechanism for DNA repair. Various repair processes are activated according to the lesion types, with double-strand breaks being the most lethal lesion to the cell as opposed to single-strand breaks [65]. Repair of these lesions can be done either through homologous recombination (HR) or non-homologous end-joining (NHEJ) [66]. In the former, either the intact chromosome or the sister chromatid serve as a template to reconstruct the missing DNA. HR is most effective in late S or G2 phase, when the sister chromatids have replicated but still attached [65]. NHEJ is more important in G1 and early S phase, but it essentially occurs throughout the cell cycle [66].

Following exposure to ionizing radiation, histone H2AX becomes phosphorylated via the ATM protein [67,68], resulting in a sharp accumulation of 53BP1 protein. This protein is involved in phosphorylating the tumor suppressor molecule p53, activating proteins essential for DNA repair, and inducing G2 checkpoint block [69-71]. Thus, G2 checkpoint induced by radiation, possibly via 53BP1, have more allocated time for repair and to escape death. G1 phase arrest also allocates more time for repair of DNA damage sustained prior to DNA synthesis.

The interval between delivered doses of radiation is very critical in the ability to effectively prevent target cells from DNA repair. It has been shown that the ability of the cell to repair DNA damage is inversely related to the dose of radiation [13]. Two doses separated in time are less cytotoxic than the sum of the two doses given at a single time, thus the benefit of hypofractionation. In addition, the more closely the two doses are applied in time, the more the resulting effects are similar to those of a large single dose. Thus, sublethal radiation tends to cause minimal DNA damage, which is then more likely to be repaired successfully [13,72,73].

### Cell death

The ultimate desired response of a cell to clinical ionizing radiation is cell death. DNA repair and cell survival are possible if the cell is exposed only to sublethal radiation dose with minimal alterations. If the limits of reparative response are exceeded, the damage is irreversible and the net result is cell death [74]. Radiation damage can activate signaling cascades that lead to programmed cell death or apoptosis [13,74]. The irradiated cell can exit permanently the cell cycle and undergo terminal differentiation. In this terminal pathway, the cell can no longer cycle and proliferate [75-77]. In other cases, the DNA damaged cell can die as it attempts to undergo mitosis, a process known as “mitotic catastrophe” [75].

### Radiation-associated prostate cancer cell gene expression

In prostate cancer cells, molecular events leading to the changes in cell cycle progression and cell survival are altered following exposure to ionizing radiation. For example, it has been shown that expression of key proteins associated with proliferation, Ki-67, and apoptosis, Bcl-2 and Bax, are dramatically altered in prostate cancer tumors following radiotherapy [78-80]. Compared to pretreatment tumors, locally recurrent prostate cancers after radiation treatment were associated with overexpression of p53 and Bcl-2 [79,81-86]. Furthermore, the overexpression of these proteins may lead to radiotherapy failure, suggesting these cellular proteins may play a part in the cellular process conferring radiation resistance.

### Radiosensitizers in prostate cancer

Understanding how cancer cells respond to ionizing radiation has enhanced the understanding of the molecular basis of radiation resistance. This in turn has led to breakthroughs in the development of strategies for increasing radiosensitivity, as well as improving the therapeutic index of radiation therapy in prostate cancer patients.

#### Radiation sensitization targets

Below is a brief review of radiation sensitization targets of interest in prostate cancer and some of the preclinical data associated with targeting these molecules (Table 1).

**Table 1:** Radiation sensitization targets

**Proteins**/**genes**	**Site of the cell**	**Cellular process**
Rad17-RFC complex	Nucleus	DNA damage sensor
9-1-1 complex	Nucleus	DNA damage sensor
MRN complex	Nucleus	DNA damage sensor
ATM (ataxia-telangiectasia mutated)	Nucleus	DNA repair
ATR (ataxia- and Rad3-related)	Nucleus	DNA repair
P53	Nucleus	DNA repair
MDM2	Nucleus	Negative regulator of p53
Chk2	Nucleus	Cell cycle
p21	Nucleus	Cell cycle
Cyclin E-CDK2 complex	Nucleus	Cell cycle
pRB	Nucleus	Cell cycle
E2F	Nucleus	Cell cycle
Cdc25A	Nucleus	Cell cycle
CDK2	Nucleus	Cell cycle
CDK4	Nucleus	Cell cycle
Cyclin B-CDK1 complex	Nucleus	Cell cycle
CAK (CDK activating kinase)	Nucleus	Cell cycle
Histone H2AX	Nucleus	Nucleosome formation
53BP1	Nucleus	P53 phosphorylation
Ki-67	Nucleus	Cellular proliferation
Bcl-2	Mitochondria	Apoptosis
Bax	Mitochondria	Apoptosis
PTEN	Cytoplasm	Apoptosis and proliferation
Akt	Cytoplasm, nucleus	Apoptosis and proliferation
PAR-4	Cytoplasm, nucleus	Apoptosis
Caspase-1	Cytoplasm	Apoptosis
Ras	Cytoplasm, nucleus	Cell growth, differentiation, survival
Cox-2	Cytoplasm, nucleus	Cell growth, differentiation, survival

### p53/MDM2

p53 expression and function in tumor cell has an important role in the cellular response to DNA damage, facilitating cell cycle arrest, and death [87]. While overexpression of p53 is associated with local recurrence of prostate cancer post-radiation, p53 gene transfer has been studied as an option to increase radiosensitivity [88]. It has been shown that combining ionizing radiation and adenoviral p53 gene therapy can induce human prostate cancer cells DU145 (p53-mutated) and PC-3 (p53-null) to be more radiosensitive [89]. Furthermore, with combination therapy, the number of apoptotic cells increased 7-fold in DU145 cells and 2-fold in PC-3 cells [89]. Radiosensitization of prostate cancer cells with adenoviral p53 gene therapy was independent of the status of p53, as the sensitization is seen in both the p53 (wild-type) human prostate cancer cell LNCaP and p53 (null) PC-3 lines [90]. In addition, *in vivo* adenovirus-mediated p53 gene therapy acts synergistically with ionizing radiation to reduce LNCaP xenograft tumor growth [91].

Mk-1775, a Wee1 kinase inhibitor has been reported to radiosensitized p53-defective human tumor cells, including PC-3 prostate cancer cells. The inhibition of Wee1 kinase produce abrogation of G2 checkpoint and as p53-defective cells cannot proceed through G1 checkpoint for repair and thus they pass directly to mitosis with DNA lesions, causing mitotic death, not apoptosis [92]. Poly (ADP-ribose) polymerase-1 (PARP1) is essential for DNA repair processes [93] but hyperactivation of PARP1 causes NAD and ATP depletions, leading in μ-calpain activation, a unique caspase-independent programmed cell death [94]. Aurora kinases are involved with cell cycle progression and regulating mitotic spindles during cell division. Of these kinases, Aurora A regulates the timing of mitotic entry and the formation of polar spindles to ensure accurate chromosome segregation. However when overexpressed, Aurora A can cause centrosome multiplication and aneuploidy, leading to carcinogenesis [95]. Furthermore, Aurora A may override the cellular machinery to arrest the development of abnormal cells, contributing to the development of radiation resistant tumors [96]. Preclinical studies of MLN8054, a specific Aurora A inhibitor that prevents phosphorylation of Thr-288, demonstrated radiosensitization in PC-3 and DU145 by enhancing DNA damage and diminishing DNA repair. Additionally when used in xenograft tumors, MLN8054 significantly reduced tumor growth, reduced tumoral angiogenesis and increased tumoral apoptotic potential [95].

MDM2, an antagonist to p53, is a key protein in the cell cycle regulation and the response of cells to ionizing radiation. MDM2 is overexpressed in many tumors including prostate cancer, and it is associated with radiation resistance. It has been shown that antisense oligonucleotides that target MDM2 can render prostate cancer cells more vulnerable to ionizing radiation independent of the p53 status, both *in vitro* and *in vivo*[97-99]. The MDM2 antisense oligonucleotide increased radiation-induced inhibitory effects on tumor growth in SCID or nude mice with LNCaP, PC3, as well as other xenografts [99].

### PTEN/Akt

The PTEN gene codes for a phosphatase in the phosphatidylinositol 3'-kinase (PI3'K)-mediated signal transduction pathway. PTEN can block pathways of proliferation and can induce apoptosis via the suppression of Akt, a serine-threonine kinase [100]. PTEN gene therapy in PC-3 cells (PTEN deleted, up-regulation of phosphorylated Akt) led to a significant decrease in cellular growth [101]. It has been further demonstrated that transfection of LNCaP prostate cells with the PTEN gene resulted in Bcl-2 downregulation [102]. Given that Bcl-2 is associated with prostate cancer cell radiation resistance [103-105] and that PTEN has tumor suppressor properties, PTEN gene therapy has been studied as a radiosensitizer. In fact, forced expression of PTEN in prostate cancer cells sensitizes cells to radiation and downregulated Bcl-2 expression in two prostate cancer cell lines that over express Bcl-2, PC-3-Bcl-2 and LNCaP. Furthermore, forced overexpression of PTEN in these prostate cancer cells potentiated a G2/M cell cycle arrest. These effects were not evident in prostate cancer cells that did not overexpress Bcl-2 [106].

### Bax/Bcl2

Stable transfection of Bcl-2 into PC-3 prostate cancer cell line rendered these cells more radiation resistant than the parent PC-3 cells [107]. In this regard, the anti-apoptotic gene Bcl-2 is associated with resistance to radiation, or at least is associated with delay of radiation-induced apoptosis in human prostate cancer cells [107]. Studies have focused on re-establishing a balance between the Bcl-2 family members in achieving radiosensitization. For example, some studies focused on selective overexpression of the pro-apoptotic gene Bax directed. Regardless of the levels of Bcl-2 protein, Bax gene therapy led to programmed-cell death [108]. Furthermore, PAR-4, another pro-apoptotic protein, is a potent modulator of NF kappa β activity and Bcl-2 protein expression. It has been found that forced over-expression of PAR-4 increases radiosensitivity in human prostate cancer cells [105]. Furthermore, Oblimersen, a phosphorothioate antisense oligonucleotide complimentary to the Bcl-2 mRNA, has being used as an inhibitor of Bcl-2 expression to enhance the therapeutic effect of radiation therapy [106].

### COX-2

Cyclooxygenase-2 (COX-2) is involved in many processes such as inflammation, proliferation, angiogenesis, carcinogenesis, and apoptosis. When overexpressed in cancer, it is associated with more aggressive biologic behavior and poor prognosis [109]. It has been reported that TNF-induced cell death and CD95-triggered apoptosis are enhanced by selective COX-2 inhibitors in previously apoptotic resistant cell lines [110]. COX-2 is constitutively expressed in both androgen-responsive LNCaP and androgen-nonresponsive PC-3 cells. The apoptotic activity of the COX-2 inhibitor, celecoxib, was studied in these prostate cancer cells. Celecoxib induces apoptosis in both cell lines. However, normal human prostate epithelial cells have low levels of COX-2, and thus do not undergo apoptosis with celecoxib [111]. In addition, celecoxib sensitized PC-3, DU145, and LNCaP prostate cancer cells to the killing effects of radiation [112]. Currently there is also *in vivo* evidence showing that celecoxib enhances tumor response to radiation in A431 human tumor xenografts in nude mice [113-115]. Thus with expression of COX-2 associated with radiation resistance and tumor aggressiveness, reduction of COX-2 expression noted to sensitize cells to radiation.

### Others

ATM is another key protein, discussed earlier, which is central in coordinating the cellular response to ionizing radiation. When PC-3 (p53-mutant) cells become infected with adenoviral vectors expressing antisense ATM RNA, their sensitivity to ionizing radiation was enhanced [116].

Other researchers are looking at transfecting adenoviruses that selectively replicate in prostate tumor cells [117]. The oncolytic adenovirus CG7870 has tumor-specific promoters driving the expression of E1A and E1B proteins. *In vitro*, combination of the adenovirus and radiation are synergistic at lower doses of radiation. *In vivo*, combination of CG7870 with radiation therapy significantly increased antitumor efficacy compared to either therapy alone [117]. Similarly *in vitro* and *in vivo* radiation sensitizing results were evident with the oncolytic adenovirus CV706 [118]. Another known aggressive phenotype of human prostate cancer is the overexpression of vascular endothelial growth factor (VEGF) and its cognate soluble receptor KDR [119]. The soluble receptor binds to VEGF and prevents its binding to its cellular receptor, while sKDR gene delivery to prostate cancer cells increased their sensitivity to ionizing radiation [120]. Furthermore, caspase-1 is a key protein involving the apoptotic pathway in prostate cancer cells [121]. Transfectants with caspase-1 have increased sensitivity to ionizing radiation [122].

The type 1 insulin-like growth factor receptor (IGF-1R) has been reported to be up-regulated in prostate cancer [123] and is speculated to play a role in cellular proliferation, cell cycle progression, and resistance to apoptosis [124]. Recently, the siRNA depletion of IGF-1R in DU145 and PC-3 cells has shown to enhanced sensitivity to radiation and as well as to DNA-damaging agents by inhibiting DNA double strand break repair [125,126], supporting the use of IGF-1R inhibitors with radiation therapy.

The A Disintegrin and Metalloprotease (ADAM) genes have been reported to play a role in cellular behavior [127], in particular prostate carcinogenesis [128-130]. Inhibition of ADAM9 in C4-2 androgen independent metastatic human prostate cancer cells by siRNA knockdown resulted in increase E-cadherin and integrins leading to sensitization to radiation and chemotherapy [131].

Signal transduction is involved in almost every important signaling pathway for cellular processes including the pathways for radiation resistance. Some molecular targets have therefore been identified for enhancing radiation effects. In one study, the radiosensitizing potential of a ribonucleotide reductase inhibitor, named Didox (DX; 3,4-Dihydroxybenzohydroxamic acid) was investigated in PC-3 cells. DX showed a significant radiosensitizing effect in p53 null prostate cancer cells by overcoming radiation induced NF kappa-β activity and Bcl-2 expression [132]. In many cancers, the proliferative phenotype is derived from enhanced activation of Ras, which is a critical protein whose overactivity is also associated with radiation resistance. In this context, blocking Ras activation with farnesyltransferase inhibitors enhanced radiosensitization of tumor cells that expressed activated Ras, in both *in vitro* studies and in xenograft models [133]. When treating prostate cancer cells with farnesyltransferase inhibitors, there was a reduction in the clonogenic survival of prostate cancer cells expressing oncogenic H-ras after irradiation [134].

Another way of enhancing radiosensitivity is through inhibition of deacetylase inhibition. The histone deacetylase (HDAC) inhibitor suberoylanilide hydroxamic acid has broad range antitumor properties [135]. Combining suberoylanilide hydroxamic acid with exposure to ionizing radiation was found to enhance radiation-induced apoptosis in DU145 [136]. Thus, the blocking of signal transduction involved in cell survival pathways is a crucial strategy for the development of radiosensitizers.

Intraprostate neuroendocrine cells have been reported to participate in prostate cancer development, progression, and resistance to conventional therapy. One neuropeptide secreted by neuroendocrine cells is neurotensin, a ligand with a high affinity to neurotensin receptor 1 (NTR1), a receptor with known stimulatory activity in several human neoplastic tissues [137-139]. NTR1 is expressed in aggressive prostate cancer cells but not in normal prostate cells [140]. NTR1 enhances DNA synthesis, cell proliferation, and survival by increasing the expression of mitogen-activated protein kinase (MAPK), phosphoinositide-3-kinase (PI3K) activation and epidermal growth factor receptor (EGFR) [141]. *In vitro* and *in vivo* studies of irradiation and SR48692, a NTR1 selective receptor antagonist, have demonstrated a reduction in EGFR phosphorylation leading to increased rates of apoptosis and reduction in xenograft tumor burden [142].

### Naturally compounds with potential radiation sensitization

Prostate cancer cells with the capacity for a more efficient repair, with a more robust cell cycle checkpoint activation and with decrease rates of apoptosis are better prepared to overcome the killing effects of radiation. Many natural compounds have been studied and reported to have significant antiproliferative and antitumorigenic effects on prostate cancer cells. There is also a trend to identify those that can potentiate the sensitivity of the cancer cells to radiation. Genistein, a naturally occurring isoflavonoid, has been reported to have potent antiproliferative effects on prostate cancer cells both *in vitro* and *in vivo*[143-146]. It is reported that genistein combined with radiation causes a significantly greater inhibition of primary tumor growth compared with genistein or radiation alone. The genistein combined with radiation is associated with a decrease in number of metastatic lymph nodes in a prostate cancer orthotopic model [146]. In DU145 cells, radiosensitivity was enhanced with even low concentrations of genistein [147]. Resveratrol (RSV), a natural polyphenol compound, was shown to enhance prostate cancer cell response to irradiation by 1) inhibiting activation of Akt, 2) enhancing activation of ATM and AMPK pathways, and 3) effecting pathways encompassing p53, p21, and p27, which are associated with early cell cycle arrest. Perturbation of these molecules result in an increase in radiation induced DNA damage and apoptosis [148]. DAB2IP is a member of GTPase-activating protein family inhibiting the Ras- mediated signal pathway and is often downregulated in prostate cancer, *i*.*e*., a potential tumor suppressor gene [149]. Loss of DAB2IP expression in prostate epithelia may lead to epithelial mesenchymal transition, which has a key role in the development of metastatic disease [150]. The loss of DAB2IP gene expression in prostate cancer cells produces an efficient DNA double-strand break repair, less cell cycle arrest and the development radiation resistance cells. Treatment with a Ras signaling pathway inhibitor, FTI-277, resulted in radiosensization of DAB2IP deficient PC-3 prostate cancer cells [151].

Defects in the apoptosis machinery have been linked to tumors being resistance to current therapeutic interventions (*e*.*g*., radiation therapy) [152]. Thus manipulation of the apoptotic pathway could prove to be beneficial. Gossypol is a natural polyphenol product from cottonseed that has been shown to be an inhibitor of the anti-apoptotic molecules, Bcl-2 and Bcl-xL [153,154], molecules associated with radiation resistance. In conjunction with radiation, (−)-Gossypol has been reported to enhance the induction of apoptosis and inhibit the growth of PC-3 xenograft tumors [155]. Next, curcumin (diferuloylmethane) is a common spice found in Asian cuisine and is the critical component of turmeric (curcuma longa) [156]. Curcumin exhibits growth inhibitory effects in a broad range of tumors [157,158]. Furthermore in combination with radiation, curcumin enhanced both radiation-induced clonogenic inhibition and apoptosis in prostate cancer cells [159]. Embelin, a natural compound with pro-apoptotic effects, *e*.*g*., inhibiting X-linked inhibitor of apoptosis protein (XIAP) while stimulating TNFα and TNF-related apoptosis-inducing ligand (TRAIL), has been shown to enhance the therapeutic efficacy of radiation therapy in PC-3 cells both *in vitro* and *in vivo*[160]. Furthermore, Selenite, another naturally occurring compound, can inhibit cell growth and induces apoptosis through p53 Ser-15 phosphorylation and caspase-mediated pathway in LNCaP cells [161]. This antiproliferative effect is associated with a decrease in the Bcl-2/Bax expression ratio and a decrease in the ratio of GSH/GSSG (essentially an oxidative state) in LAPC-4 prostate cancer cells. Furthermore, both LAPC-4 and DU145 cells showed increased radiosensitivity when pre-treated with selenite [162].

Oxidative stress leading to higher levels of reactive oxygen species (ROS) is a common hallmark of cancer cells, which can stimulate cellular proliferation and cause cellular injury. Parthenolide, a sesquiterpene lactone derived from the herbal medicine feverfew, has been reported to produce a selective radiosensitization in prostate cancer cells and not effect normal prostate epithelial cells by activation of NADPH oxidase, which is an important source of ROS in prostate cancer cells and by suppressing antioxidants agents. Furthermore, parthenolide decreases radiation-induced ROS in normal prostate epithelial cells, suggesting that the intracellular redox status maybe a way to achieve selective drug targeting [163].

Hypoxia is a feature of many human tumors and has been implicated as an important biologically modulator of clinical behavior and treatment response in prostate cancer. Up-regulation of hypoxia-inducible factor (HIF) signaling has been reported to be an independent predictor of biochemical failure in prostate cancer patients treat with either surgery or radiotherapy [164]. Furthermore, a recent study of 247 patients with localized prostate cancer treated with high-dose external beam radiation therapy demonstrated that tumor hypoxia was associated with early biochemical relapse and local recurrence [165]. Thus an opportunity exists to alter the intratumoral balance of oxygen during radiation treatment in hopes of producing DNA damaging free radicals.

### Clinical trials of radiation sensitization targets

With the deluge of targeted agents over the past decade, it is rather surprising that limited studies have been reported with these agents or any agent deemed to sensitize prostatic tumors to the effects of radiation therapy. Since 2000, only three such trials have been reported in the English literature (two using targeted agents, one using natural products). In a phase I/II study by Joensuu *et al*. daily administration of 250 mg of gefitinib given concurrently with three-dimensional conformal radiation therapy for patients with nonmetastatic prostate cancer was well tolerated. At a median follow-up of 38 months, 97% of patient biochemically had no evidence of tumor recurrence [166]. In a small phase II study of 18 patients with high-risk prostate cancer, patients were treated with maximum androgen blockade, bevacizumab (10 mg/kg every 2 week then 15 mg/kg every 3 week for 12 additional weeks) with concomitant radiation (77.9 Gy delivered by intensity modulated radiation therapy) to the prostate. The regimen was well tolerated with no increase in acute toxicities and a slight increase in late toxicities related to proctitis and cystitis [167]. In a small pilot study reported by Ahmad *et al*., 42 patients with prostate cancer were randomly assigned to receive 200 mg of soy isoflavone or placebo daily for six months beginning on the first day of radiation therapy. Total dose of radiation was 77.5 Gy. The authors did not comment on oncologic outcomes, but it was reported that the therapy was well tolerated and perhaps lessen adverse effects seen with radiation therapy [168].

The importance of incorporating novel therapies in hopes of sensitizing prostatic tumors to radiation is best illustrated when ADT was combined with radiation therapy. Preclinical studies demonstrated that ADT could induce apoptosis and inhibit angiogenesis in addition to potentiating the effects of radiation therapy [169]. These preclinical studies subsequently were translated into a clinical therapeutic advantage in men with high-risk localized prostate cancer [170,171]. Fourteen trials using radiosensitizing agents are in progress or recently completed (Table 2) in men with high-risk prostate cancer treated with radiation therapy. A more concerted effort must be made to bring promising targeted therapeutics to clinical trial in order to determine treatment efficacy.

**Table 2:** Accruing clinical trials combining radiosensitizing agents with radiation therapy

**Trial description**	**Sponsor**	**Status**
Sunitinib with hormonal ablation in patients with localized prostate cancer	MD Anderson Cancer Center	Ongoing, not recruiting
SU5416 with hormonal ablation in patients with localized prostate cancer	University of Chicago	Unknown
Everolimus with hormonal ablation in patients with high risk localized prostate cancer	University of Michigan	Not yet open
TAK-700 with hormonal ablation in patients with high risk localized prostate cancer	Radiation Therapy Oncology Group	Recruiting
Everolimus with hormonal ablation in patients with high risk locally advanced prostate cancer	Centre Val d’Aurelle – Paul Lamarque	Recruiting
Bevacizumab with hormonal ablation in patients with high risk localized prostate cancer	Benaroya Research Institute	Completed
Everolimus for salvage treatment of biochemical recurrence after prostatectomy	Abramson Cancer Center of the University of Pennsylvania	Recruiting
IL-12 gene therapy	Baylor College of Medicine	Completed
ProstAtak™ in patients with localized prostate cancer	Advantagene, Inc.	Recruiting
Isoflavones in patients with localized prostate cancer	Barbara Ann Karmanos Cancer Institute	Completed
Eflornithine and Bicalutamide Compared With Eflornithine Alone, Bicalutamide Alone, and No Neoadjuvant Therapy in Treating Patients With Localized Prostate Cancer	University of Alabama	Completed
Selenomethionine in patients with localized prostate cancer	Roswell Park Cancer Center	Withdrawn
R-Flubiprofen in patients with high risk localized prostate cancer	Myrexis Inc.	Unknown
Panobinostat in patients with localized prostate cancer	Novartis Pharmaceuticals	Completed

## Conclusions

Radiation therapy continues to be one of the more popular treatment options for the definitive treatment of localized prostate cancer. Recent research has helped understand and identify maximal tolerated radiation doses needed to treat prostate cancer. Thus, continuing to increase radiation doses may not show a clinical benefit and may be fraught with toxicity. Another way to improve the therapeutic efficacy of radiation is to sensitize the cells to the effects of radiation at current or even lower radiation doses. Different strategies are being pursued at this time to achieve these goals. Further development in this field will come as we gain more understanding of the cellular pathways leading to radiation resistance and how to best selectively block these pathways. Though limited clinical trials in prostate cancer patients support radiosensitizing agents, the field holds promise and should be aggressively explored further.

## Abbreviations

ATM: Ataxia-telangiectesia mutated; CAK: CDK activating kinase; HR: Homologous recombination; NHEJ: Non-homologous end-joining; PARP1: Poly (ADP-ribose) polymerase-1; PI3’K: Phosphatidylinositol 3'-kinase; COX-2: Cyclooxygenase-2; VEGF: Vascular endothelial growth factor; IGF-1R: Type 1 insulin-like growth factor receptor; ADAM: A Disintegrin and Metalloprotease; HDAC: Histone deacetylase; NTR1: Neurotensin receptor 1; MAPK: Mitogen-activated protein kinase; EGFR: Epidermal growth factor receptor; RSV: Resveratrol; XIAP: X-linked inhibitor of apoptosis protein; TRAIL: TNF-related apoptosis-inducing ligand; HIF: Hypoxia-inducible factor.

## Competing interests

The authors declare that they have no competing interests.

## Authors’ contributions

DAPA – performed literature search and drafted the manuscript. MM – performed literature search and drafted the manuscript. CJR – supervised the project and revised drafted manuscript. All authors read and approved the final manuscript.

## Pre-publication history

The pre-publication history for this paper can be accessed here:

http://www.biomedcentral.com/1471-2490/13/4/prepub

## References

[B1] Siegel R Naishadham D Jemal A Cancer statistics 2012 CA Cancer J Clin 2012 62 10 29 10.3322/caac.20138 22237781

[B2] National Cancer Institute SEER cancer statistics review 1975–2008. Lifetime risk (percent) of being diagnosed with cancer by site and race/ethnicity: males, 17 SEER areas, 2006–2008 (Table 1.15) And females, 17 SEER areas, 2006–2008 (Table 1.16) 2011 Accessed at http://seer.cancer.gov/csr/1975_2008/results_merged/topic_lifetime_risk_diagnosis.pdf on August 28, 2012

[B3] Merrill RM Brawley OW Prostate cancer incidence and mortality rates among white and black men Epidemiology 1997 8 126 131 10.1097/00001648-199703000-00001 9229202

[B4] D'Amico AV Vogelzang NJ Prostate brachytherapy: increasing demand for the procedure despite the lack of standardized quality assurance and long term outcome data Cancer 1999 86 9 1632 1634 10.1002/(SICI)1097-0142(19991101)86:9<1632::AID-CNCR2>3.0.CO;2-5 10547533

[B5] Zelefsky MJ Fuks Z Leibel SA Intensity-modulated radiation therapy for prostate cancer Semin Radiat Oncol 2002 12 3 229 237 Review 10.1053/srao.2002.00000 12118388

[B6] Ravery V Brachytherapy versus radical prostatectomy BJU Int 2001 87 141 143 11167631 10.1046/j.1464-410x.2001.00026.x

[B7] Shipley WU Verhey LJ Munzenrider JE Suit HD Urie MM McManus PL Advanced prostate cancer: the results of a randomized comparative trial of high dose irradiation boosting with conformal protons compared with conventional dose irradiation using photons alone Int J Radat Oncol Biol Phys 1995 32 3 12 10.1016/0360-3016(95)00063-5 7721636

[B8] Kuban DA El-Mahdi AM Schellhammer PF Effect of local tumor control on distant metastasis and survival in prostatic adenocarcinoma Urol 1987 30 420 426 10.1016/0090-4295(87)90372-4 3118547

[B9] Zagars GK von Eschenbach AC Johnson DE Oswald MJ Stage C adenocarcinoma of the prostate. An analysis of 551 patients treated with external beam radiation Cancer 1987 60 1489 1499 10.1002/1097-0142(19871001)60:7<1489::AID-CNCR2820600715>3.0.CO;2-9 3113715

[B10] Pollack A Zagars GK Starkschall G Childress CH Kopplin S Boyer AL Conventional vs conformal radiotherapy for prostate cancer. Preliminary results of Dosimetry and acute toxicity Int J Radiat Oncol Biol Phys 1996 34 555 564 10.1016/0360-3016(95)02103-5 8621278

[B11] Bagshaw MA Kaplan ID Cox RC Prostate cancer. Radiation therapy for localized disease Cancer 1993 47 929 952 10.1002/1097-0142(19930201)71:3+<939::aid-cncr2820711409>3.0.co;2-0 8428344

[B12] Pollack A Zagars GK Smith LG Lee JJ von Eschenbach AC Antolak JA Starkschall G Rosen I Preliminary results of a randomized radiotherapy dose-escalation study comparing 70 Gy with78 Gy for prostate cancer JCO 2000 18 3904 3911 10.1200/JCO.2000.18.23.3904 11099319

[B13] Smith RP McKenna WG The Basics of Radiation Therapy Clinical Oncology 2004 3

[B14] Perez CA Brady L Principles and practice of radiation oncology 1997 3 Philadelphia: Lippincott Williams & Wilkins

[B15] Johns HE The physicist in cancer treatment and detection Int J Radiat Oncol Biol Phys 1981 7 801 808 10.1016/0360-3016(81)90477-6 6793544

[B16] Radford IR Evidence for a general relationship between the induced level of DNA double-strand breakage and cell-killing after X-irradiation of mammalian cells Int J Radiat Biol Relat Stud Phys Chem Med 1986 49 611 620 3485603 10.1080/09553008514552861

[B17] Nunez MI McMillan TJ Valenzuela MT de Almodovar JM R Pedraza V Relationship between DNA damage, rejoining and cell killing by radiation in mammalian cells Radiother Oncol 1996 39 155 165 10.1016/0167-8140(96)01732-X 8735483

[B18] Lunec J Introductory review: involvement of ADP-ribosylation in cellular recovery from some forms of DNA damage Br J Cancer 1984 6 13 18 PMC2149140 6320850

[B19] Dikomey E Dahm-Daphi J Brammer I Martensen R Kaina B Correlation between cellular radiosensitivity and non-repaired double-strand breaks studied in nine mammalian cell lines Int J Radiat Biol 1998 73 269 278 10.1080/095530098142365 9525255

[B20] Kemp LM Sedgwick SG Jeggo P AX-ray sensitive mutants of Chinese hamster ovary cells defective in double-strand break rejoining Mutat Res 1984 132 189 196 10.1016/0167-8817(84)90037-3 6513971

[B21] Munro TR The relative radiosensitivity of the nucleus and cytoplasm of Chinese hamster fibroblasts Radiat Res 1970 42 451 470 10.2307/3572962 5463516

[B22] Munro TR The relative radiosensitivity of the nucleus and cytoplasm of Chinese hamster fibroblasts Exp Cell Res 1960 20 613 10.1016/0014-4827(60)90138-5 5463516

[B23] Walters R Hofer K Harris C Radionuclide toxicity in cultured mammalian cells: Elucidation of the primary site of radiation damage Ann Top Radiat Res Q 1977 12 389 565271

[B24] Brenner DJ Ward JF Constraints on energy deposition and target size of multiply damaged sites associated with DNA double-strand breaks Int J Radiat Biol 1992 61 737 748 10.1080/09553009214551591 1351522

[B25] Jonah CD A short history of the radiation chemistry of water Radiat Res 1995 144 141 147 10.2307/3579253 7480640

[B26] Howard-Flanders P Moore D The time interval after pulsed irradiation within which injury in bacteria can be modified by dissolved oxygen. I. A Search for an effect of oxygen 0.02 seconds after pulsed irradiation Radiat Res 1958 9 422 437 10.2307/3570768 13591515

[B27] Ward JF Biochemistry of DNA lesions Radiat Res 1985 8 103 111 3867077

[B28] Goodhead D Hagen U, Jung P, Streffer C Physics of radiation action: Microscopic features that determine biological consequences10th International Congress of Radiation Research 1995 Wurzburg, Germany: Congress Lectures 43

[B29] Molls M Stadler P Becker A Feldmann HJ Dunst J Relevance of oxygen in radiation oncology. Mechanisms of action, correlation to low hemoglobin levels Strahlenther Onkol 1998 174 13 16 9879341

[B30] Steel G Adama C Peckham M The Biological Basis of Radiotherapy 1983 Amsterdam: Elsevier Science

[B31] Kaplan HS Historic milestones in radiobiology and radiation therapy Semin Oncol 1979 6 479 489 119321

[B32] Wilson GD Radiation and the cell cycle, revisited Cancer Metastasis Rev 2004 23 209 225 15197324 10.1023/B:CANC.0000031762.91306.b4

[B33] Griffiths DJ Barbet NC McCready S Lehmann AR Carr AM Fission yeast rad17: A homologue of budding yeast RAD24 that shares regions of sequence similarity with DNA polymerase accessory proteins EMBO J 1995 14 5812 5823 8846774 10.1002/j.1460-2075.1995.tb00269.x PMC394699

[B34] St Onge RP Udell CM Casselman R Davey S The human G2 checkpoint control protein hRAD9 is a nuclear phosphoprotein that forms complexes with hRAD1 and hHUS1 Mol Biol Cell 1999 10 1985 1995 10359610 10.1091/mbc.10.6.1985 PMC25401

[B35] Maser RS Monsen KJ Nelms BE Petrini JH hMre11 and hRad50 nuclear foci are induced during the normal cellular response to DNA double-strand breaks Mol Cell Biol 1997 17 6087 6096 9315668 10.1128/mcb.17.10.6087 PMC232458

[B36] Lydall D Weinert T Yeast checkpoint genes in DNA damage processing: Implications for repair and arrest Science 1995 270 1488 1491 10.1126/science.270.5241.1488 7491494

[B37] Tauchi H Kobayashi J Morishima K van Gent DC Shiraishi T Verkaik NS van Heems D Ito E Nakamura A Sonoda E Takata M Takeda S Matsuura S Komatsu K Nbs1 is essential for DNA repair by homologous recombination in higher vertebrate cells Nature 2002 420 93 98 10.1038/nature01125 12422221

[B38] Kim ST Lim DS Canman CE Kastan MB Substrate specificities and identification of putative substrates of ATM kinase family members J Biol Chem 1999 274 37538 37543 10.1074/jbc.274.53.37538 10608806

[B39] Wright JA Keegan KS Herendeen DR Bentley NJ Carr AM Hoekstra MF Concannon P Protein kinase mutants of human ATR increase sensitivity to UV and ionizing radiation and abrogate cell cycle checkpoint control Proc Natl Acad Sci USA 1998 95 7445 7450 10.1073/pnas.95.13.7445 9636169 PMC22645

[B40] Abraham RT Checkpoint signaling: epigenetic events sound the DNA strand-breaks alarm to the ATM protein kinase Bioessays 2003 25 627 630 10.1002/bies.10310 12815717

[B41] Bakkenist CJ Kastan MB DNA damage activates ATM through intermolecular autophosphorylation and dimer dissociation Nature 2003 421 499 506 10.1038/nature01368 12556884

[B42] Morgan DO Principles of CDK Regulation Nature 1995 374 131 134 10.1038/374131a0 7877684

[B43] Nigg EA Cyclin-dependent protein kinases: Key regulators of the eukaryotic cell cycle Bioassays 1995 17 471 480 10.1002/bies.950170603 7575488

[B44] Lopez-Saez JF la TC D Pincheira J Gimenez-Martin G Cell Proliferation and Cancer Histol Histopathol 1998 13 1197 1214 9810511 10.14670/HH-13.1197

[B45] Zetterberg A Larsson O Wiman KG What is the restriction point? Curr Opin Cell Biol 1995 7 835 842 10.1016/0955-0674(95)80067-0 8608014

[B46] Terasima T Tolmach LJ X-ray sensitivity and DNA synthesis in synchronous populations of HeLa cells Science 1963 140 490 492 10.1126/science.140.3566.490 13980636

[B47] Terasima T Tolmach LJ Variations in several responses of HeLa cells to x-irradiation during the division cycle Biophys J 1963 3 11 33 10.1016/S0006-3495(63)86801-0 13980635 PMC1366421

[B48] Sinclair WK Cyclic x-ray responses in mammalian cells in vitro Radiat Res 1968 33 620 643 10.2307/3572419 4867897

[B49] Biade S Stobbe CC Chapman JD The intrinsic radiosensitivity of some human tumor cells throughout their cell cycles Radiat Res 1997 147 416 421 10.2307/3579497 9092920

[B50] Zhang Y Xiong Y A p53 amino-terminal nuclear export signal inhibited by DNA damage-induced phosphorylation Science 2001 292 1910 1915 10.1126/science.1058637 11397945

[B51] Shieh SY Ahn J Tamai K Taya Y Prives C The human homologs of checkpoint kinases Chk1 and Cds1 (Chk2) phosphorylate p53 at multiple DNA damage-inducible sites Genes Dev 2000 14 289 300 10673501 PMC316358

[B52] Sherr CJ Roberts JM CDK inhibitors: Positive and negative regulators of G1-phase progression Genes Dev 1999 13 1501 1512 10.1101/gad.13.12.1501 10385618

[B53] Harbour JW Dean DC The Rb/E2F pathway: expanding roles and emerging paradigms Genes Dev 2000 14 2393 2409 10.1101/gad.813200 11018009

[B54] Beijersbergen RL Bernards R Cell cycle regulation by the retinoblastoma family of growth inhibitory proteins Biochim Biophys Acta 1996 1287 103 120 8672526 10.1016/0304-419x(96)00002-9

[B55] Mailand N Falck J Lukas C Syljuasen RG Welcker M Bartek J Lukas J Rapid destruction of human Cdc25A in response to DNA damage Science 2000 288 1425 1429 10.1126/science.288.5470.1425 10827953

[B56] Schneiderman MH Schneiderman GS Rusk CM A cell kinetic method for the mitotic selection of treated G2 cells Cell Tissue Kinet 1983 16 41 49 6825155

[B57] Schneiderman MH Schneiderman GS G2 Cells: progression delay and survival Radiat Res 1984 98 389 396 10.2307/3576246 6539484

[B58] Friedberg EC DNA damage and repair Nature 2003 421 436 440 10.1038/nature01408 12540918

[B59] Valerie K Povirk LF Regulation and mechanisms of mammalian double-strand break repair Oncogene 2003 22 5792 5812 10.1038/sj.onc.1206679 12947387

[B60] Sarkaria JN Tibbetts RS Busby EC Kennedy AP Hill DE Abraham RT Inhibition of phosphoinositide 3-kinase related kinases by the radiosensitizing agent wortmannin Cancer Res 1998 58 4375 4382 9766667

[B61] Falck J Mailand N Syljuasen RG Bartek J Lukas J The ATM-Chk2-Cdc25A checkpoint pathway guards against radioresistant DNA synthesis Nature 2001 410 842 847 10.1038/35071124 11298456

[B62] Kaldis P The cdk-activating kinase (CAK): from yeast to mammals Cell Mol Life Sci 1999 55 284 296 10.1007/s000180050290 10188587 PMC11146862

[B63] Parker LL Atherton-Fessler S Piwnica-Worms H p107wee1 is a dual-specificity kinase that phosphorylates p34cdc2 on tyrosine 15 Proc Natl Acad Sci USA 1992 89 2917 2921 10.1073/pnas.89.7.2917 1372994 PMC48774

[B64] Nyberg KA Michelson RJ Putnam CW Weinert TA Toward maintaining the genome: DNA damage and replication checkpoints Annu Rev Genet 2002 36 617 656 10.1146/annurev.genet.36.060402.113540 12429704

[B65] Haber JE Partners and pathways repairing a double-strand break Trends Genet 2000 16 259 264 10.1016/S0168-9525(00)02022-9 10827453

[B66] Takata M Sasaki MS Sonoda E Morrison C Hashimoto M Utsumi H Yamaguchi-Iwai Y Shinohara A Takeda S Homologous recombination and non-homologous end-joining pathways of DNA double-strand break repair have overlapping roles in the maintenance of chromosomal integrity in vertebrate cells EMBO J 1998 17 5497 5508 10.1093/emboj/17.18.5497 9736627 PMC1170875

[B67] Rogakou EP Pilch DR Orr AH Ivanova VS Bonner WM DNA double-stranded breaks induce histone H2AX phosphorylation on serine 139 J Biol Chem 1998 273 5858 5868 10.1074/jbc.273.10.5858 9488723

[B68] Burma S Chen BP Murphy M Kurimasa A Chen DJ ATM phosphorylates histone H2AX in response to DNA double-strand breaks J Biol Chem 2001 276 42462 42467 10.1074/jbc.C100466200 11571274

[B69] Wang B Matsuoka S Carpenter PB Elledge SJ 53BP1, a mediator of the DNA damage checkpoint Science 2002 298 1435 1438 10.1126/science.1076182 12364621

[B70] DiTullio RA Jr Mochan TA Venere M 53BP1 functions in an ATM-dependent checkpoint pathway that is constitutively activated in human cancer Nat Cell Biol 2002 4 998 1002 10.1038/ncb892 12447382

[B71] Kao GD McKenna WG Guenther MG Muschel RJ Lazar MA Yen TJ Histone deacetylase 4 interacts with 53BP1 to mediate the DNA damage response J Cell Biol 2003 160 1017 1027 10.1083/jcb.200209065 12668657 PMC2172769

[B72] Elkind M Sutton H Radiation response of mammalian cells grown in culture I. Repair of x-ray damage in surviving Chinese hamster cells Radiation Res 1960 13 556 10.2307/3570945 13726391

[B73] Belli JA Dicus GJ Bonte FJ Radiation response of mammalian tumor cells I. Repair of sublethal damage in vivo J Natl Cancer Inst 1967 38 673 682 6025764

[B74] Majno G Joris I Apoptosis, oncosis, and necrosis. An overview of cell death Am J Pathol 1995 146 3 15 7856735 PMC1870771

[B75] Favaudon V Cell cycle regulation and radiation-induced cell death Cancer Radiother 2000 4 355 368 10.1016/S1278-3218(00)00009-3 11098223

[B76] Chang WP Little JB Delayed reproductive death as a dominant phenotype in cell clones surviving X-irradiation Carcinogenesis 1992 13 923 928 10.1093/carcin/13.6.923 1600612

[B77] Radford IR Murphy TK Radiation response of mouse lymphoid and myeloid cell lines part III. Different signals can lead to apoptosis and may influence sensitivity to killing by DNA double-strand breakage Int J Radiat Biol 1994 65 229 239 10.1080/09553009414550261 7907120

[B78] Pollack A Cowen D Troncoso P Zagars GK von Eschenbach AC Meistrich ML McDonnell T Molecular markers of outcome after radiotherapy in patients with prostate carcinoma: Ki-67, bcl-2, bax, and bcl-x Cancer 2003 97 7 1630 1638 10.1002/cncr.11230 12655519

[B79] Rakozy C Grignon DJ Sarkar FH Sakr WA Littrup P Forman J Expression of Bcl-2, p53, and p21 in benign and malignant prostatic tissue before and after radiation therapy Mod Pathol 1998 11 9 892 899 9758370

[B80] Rosser CJ Reyes AO Vakar-Lopez F Levy LB Kuban DA Hoover DC Lee AK Pisters LL Bcl-2 is significantly overexpressed in localized radio-recurrent prostate carcinoma, compared with localized radio-naive prostate carcinoma Int J Radiat Oncol Biol Phys 2003 56 1 1 6 10.1016/S0360-3016(02)04468-1 12694817

[B81] Cheng L Sebo TJ Cheville JC Pisansky TM Slezak J Bergstralh EJ Pacelli A Neumann RM Zincke H Bostwick DG p53 Protein overexpression is associated with increased cell proliferation in patients with locally recurrent prostate carcinoma after radiation therapy Cancer 1999 85 6 1293 1299 10.1002/(SICI)1097-0142(19990315)85:6<1293::AID-CNCR11>3.0.CO;2-O 10189134

[B82] Grossfeld GD Olumi AF Connolly JA Chew K Gibney J Bhargava V Waldman FM Carroll PR Locally recurrent prostate tumors following either radiation therapy or radical prostatectomy have changes in Ki-67 labeling index, p53 and bcl-2 immunoreactivity J Urol 1998 159 5 1437 1443 10.1097/00005392-199805000-00004 9554329

[B83] Huang A Gandour-Edwards R Rosenthal SA Siders DB Deitch AD White RW p53 And Bcl-2 immunohistochemical alterations in prostate cancer treated with radiation therapy Urology 1998 51 2 346 351 10.1016/S0090-4295(97)00636-5 9495727

[B84] Prendergast NJ Atkins MR Schatte EC Paulson DF Walther PJ p53 Immunohistochemical and genetic alterations are associated at high incidence with post-irradiated locally persistent prostate carcinoma J Urol 1996 155 5 1685 1692 10.1016/S0022-5347(01)66165-2 8627854

[B85] Rakozy C Grignon DJ Li Y Gheiler E Gururajanna B Pontes JE Sakr W Wood DP Jr Sarkar FH p53 Gene alterations in prostate cancer after radiation failure and their association with clinical outcome: a molecular and immunohistochemical analysis Pathol Res Pract 1999 195 3 129 135 10.1016/S0344-0338(99)80024-7 10220791

[B86] Scherr DS Vaughan ED Jr Wei J Chung M Felsen D Allbright R Knudsen BS Bcl-2 and p53 expression in clinically localized prostate cancer predicts response to external beam radiotherapy J Urol 1999 162 2 503 10.1097/00005392-199907000-00003 10379729

[B87] Vousden KH Lu X Live or let die: the cell's response to p53 Nat Rev Cancer 2002 2 594 604 10.1038/nrc864 12154352

[B88] Pisters LL Pettaway CA Troncoso P McDonnell TJ Stephens LC Wood CG Do KA Brisbay SM Wang X Hossan EA Evans RB Soto C Jacobson MG Parker K Merritt JA Steiner MS Logothetis CJ Evidence that transfer of functional p53 protein results in increased apoptosis in prostate cancer Clin Cancer Res 2004 10 8 2587 2593 I added a better reference here 10.1158/1078-0432.CCR-03-0388 15102659

[B89] Sasaki R Shirakawa T Zhang ZJ Tamekane A Matsumoto A Sugimura K Matsuo M Kamidono S Gotoh A Additional gene therapy with Ad5CMV-p53 enhanced the efficacy of radiotherapy in human prostate cancer cells Int J Radiat Oncol Biol Phys 2001 51 5 1336 1345 10.1016/S0360-3016(01)01803-X 11728695

[B90] Colletier PJ Ashoori F Cowen D Meyn RE Tofilon P Meistrich ME Pollack A Adenoviral-mediated p53 transgene expression sensitizes both wild-type and null p53 prostate cancer cells in vitro to radiation Int J Radiat Oncol Biol Phys 2000 48 5 1507 1512 10.1016/S0360-3016(00)01409-7 11121656

[B91] Cowen D Salem N Ashoori F Meyn R Meistrich ML Roth JA Pollack A Prostate cancer radiosensitization in vivo with adenovirus-mediated p53 gene therapy Clin Cancer Res 2000 6 11 4402 4408 11106260

[B92] Bridges KA Hirai H Buser CA Brooks C Liu H Buchholz TA Molkentine JM Mason KA Meyn RE MK-1775, a novel Wee1 kinase inhibitor, radiosensitizes p53-defective human tumor cells Clin Cancer Res 2011 17 17 5638 5648 10.1158/1078-0432.CCR-11-0650 21799033 PMC3167033

[B93] Noël G Giocanti N Fernet M Mégnin-Chanet F Favaudon V Poly (ADP-ribose) polymerase (PARP-1) is not involved in DNA double strand break recovery BMC Cell Biol 2003 4 7 10.1186/1471-2121-4-7 12866953 PMC179890

[B94] Tagliarino C Pink J Reinicke K Simmers S Wuerzberger-Davis S Boothman D Mu-calpain activation in β-lapachone-mediated apoptosis Cancer Biol Ther 2003 2 141 152 12750552 10.4161/cbt.2.2.237

[B95] Moretti L Niermann K Schleicher S Giacalone NJ Varki V Kim KW Kopsombut P Jung DK Lu B MLN8054, a small molecule inhibitor of aurora kinase a, sensitizes androgen-resistant prostate cancer to radiation Int J Radiat Oncol Biol Phys 2011 80 4 1189 1197 10.1016/j.ijrobp.2011.01.060 21514073

[B96] Guan Z Wang XR Zhu XF Aurora-A, a negative prognostic marker, increases migration and decreases radiosensitivity in cancer cells Cancer Res 2007 67 10436 10444 10.1158/0008-5472.CAN-07-1379 17974987

[B97] Bianco R Ciardiello F Tortora G Chemosensitization by antisense oligonucleotides targeting MDM2 Curr Cancer Drug Targets 2005 5 1 51 56 10.2174/1568009053332681 15720189

[B98] Zhang R Wang H Agrawal S Novel antisense anti-MDM2 mixed-backbone oligonucleotides: proof of principle, in vitro and in vivo activities, and mechanisms Curr Cancer Drug Targets 2005 5 1 43 49 10.2174/1568009053332663 15720188

[B99] Zhang Z Wang H Prasad G Li M Yu D Bonner JA Agrawal S Zhang R Radiosensitization by Antisense Anti-MDM2 Mixed-Backbone Oligonucleotide in in Vitro and in Vivo Human Cancer Models Clin Cancer Res 2004 10 4 1263 1273 10.1158/1078-0432.CCR-0245-03 14977824

[B100] Davies MA Koul D Dhesi H Regulation of Akt PKB activity, cellular growth, and apoptosis in prostate carcinoma cells by MMAC PTEN Cancer Res 1999 59 2551 2556 10363971

[B101] Tanaka M Rosser CJ Grossman HB PTEN gene therapy induces growth inhibition and increases efficacy of chemotherapy in prostate cancer Cancer Detect Prev 2005 29 2 170 174 10.1016/j.cdp.2004.07.006 15829377

[B102] Rosser CJ Tanaka M Pisters LL Tanaka N Levy LB Hoover DC Grossman HB McDonnell TJ Kuban DA Meyn RE Adenoviral-mediated PTEN Transgene Expression Sensitizes Bcl-2-Expressing Prostate Cancer Cells to Radiation Cancer Gene Ther 2004 11 4 273 279 10.1038/sj.cgt.7700673 14765130

[B103] Kyprianou N King ED Bradbury D Rhee JG Bcl-2 Over-Expression Delays Radiation-Induced Apoptosis without Affecting the Clonogenic Survival of Human Prostate Cancer Cells Int J Cancer 1997 70 3 341 348 10.1002/(SICI)1097-0215(19970127)70:3<341::AID-IJC16>3.0.CO;2-I 9033638

[B104] Lowe SL Rubinchik S Honda T McDonnell TJ Dong JY Norris JS Prostate-specific expression of Bax delivered by an adenoviral vector induces apoptosis in LNCaP prostate cancer cells Gene Ther 2001 8 18 1363 1371 10.1038/sj.gt.3301531 11571575

[B105] Chendil D Das A Dey S Mohiuddin M Ahmed MM Par-4, a Pro-apoptotic gene, inhibits radiation-induced NF kappa B activity and Bcl-2 expression leading to induction of radiosensitivity in human prostate cancer cells PC-3 Cancer Biol Ther 2002 1 2 152 160 12170775 10.4161/cbt.61

[B106] Anai S Goodison S Shiverick K Iczkowski K Tanaka M Rosser CJ Combination of PTEN gene therapy and radiation inhibits the growth of human prostate cancer xenografts Hum Gene Ther 2006 17 10 975 984 10.1089/hum.2006.17.975 16984224

[B107] Anai S Goodison S Shiverick K Hirao Y Brown BD Rosser CJ Knock-down of Bcl-2 by antisense oligodeoxynucleotides induces radiosensitization and inhibition of angiogenesis in human PC-3 prostate tumor xenografts Mol Cancer Ther 2007 6 1 101 111 10.1158/1535-7163.MCT-06-0367 17237270

[B108] Honda T Kagawa S Spurgers KB Gjertsen BT Roth JA Fang B Lowe SL Norris JS Meyn RE McDonnell TJ A recombinant adenovirus expressing wild-type Bax induces apoptosis in prostate cancer cells independently of their Bcl-2 status and androgen sensitivity Cancer Biol Ther 2002 1 2 163 167 12170776 10.4161/cbt.63

[B109] Choy H Milas L Enhancing Radiotherapy with Cyclooxygenase-2 Enzyme Inhibitors: a Rational advance? J Natl Cancer Inst 2003 95 19 1440 1452 10.1093/jnci/djg058 14519750

[B110] Totzke G Schulze-Osthoff K Janicke RU Cyclooxygenase-2 (COX-2) inhibitors sensitize tumor cells specifically to death receptor-induced apoptosis independently of COX-2 inhibition Oncogene 2003 22 39 8021 8030 10.1038/sj.onc.1206837 12970750

[B111] Hsu AL Ching TT Wang DS Song X Rangnekar VM Chen CS The cyclooxygenase-2 inhibitor celecoxib induces apoptosis by blocking Akt activation in human prostate cancer cells independently of Bcl-2 J Biol Chem 2000 275 15 11397 11403 10.1074/jbc.275.15.11397 10753955

[B112] Anai S Tanaka M Shiverick KT Kim W Takada S Boehlein S Goodison S Mizokami A Rosser CJ Increased expression of cyclooxygenase-2 correlates with resistance to radiation in human prostate adenocarcinoma cells J Urol 2007 177 5 1913 1917 10.1016/j.juro.2007.01.019 17437847

[B113] Nakata E Mason KA Hunter N Husain A Raju U Liao Z Ang KK Milas L Potentiation of tumor response to radiation or chemoradiation by selective cyclooxygenase-2 enzyme inhibitors Int J Radiat Oncol Biol Phys 2004 58 369 375 10.1016/j.ijrobp.2003.09.061 14751505

[B114] Milas L Cyclooxygenase-2 (COX-2) enzyme inhibitors and radiotherapy: preclinical basis Am J Clin Oncol 2003 26 4 S66 S69 10.1097/00000421-200308002-00006 12902859

[B115] Davis TW Hunter N Trifan OC Milas L Masferrer JL COX-2 inhibitors as radiosensitizing agents for cancer therapy Am J Clin Oncol 2003 26 4 S58 S61 10.1097/00000421-200308002-00004 12902857

[B116] Fan Z Chakravarty P Alfieri A Pandita TK Vikram B Guha C Adenovirus-mediated antisense ATM gene transfer sensitizes prostate cancer cells to radiation Cancer Gene Ther 2000 7 10 1307 1314 10.1038/sj.cgt.0243 11059687

[B117] Dilley J Reddy S Ko D Nguyen N Rojas G Working P Yu DC Oncolytic adenovirus CG7870 in combination with radiation demonstrates synergistic enhancements of antitumor efficacy without loss of specificity Cancer Gene Ther 2005 12 8 715 722 15832172 10.1038/sj.cgt.7700835

[B118] Chen Y DeWeese T Dilley J Zhang Y Li Y Ramesh N Lee J Pennathur-Das R Radzyminski J Wypych J Brignetti D Scott S Stephens J Karpf DB Henderson DR Yu DC CV706, a prostate cancer-specific adenovirus variant, in combination with radiotherapy produces synergistic antitumor efficacy without increasing toxicity Cancer Res 2001 61 14 5453 5460 11454691

[B119] Qi L Robinson WA Brady BM Glode LM Migration and invasion of human prostate cancer cells is related to expression of VEGF and its receptors Anticancer Res 2003 23 5a 3917 3922 14666697

[B120] Kaliberov SA Kaliberova LN Buchsbaum DJ Combined ionizing radiation and sKDR gene delivery for treatment of prostate carcinomas Gene Ther 2005 12 5 407 417 10.1038/sj.gt.3302432 15616600

[B121] Guo Y Kyprianou N Restoration of transforming growth factor beta signaling pathway in human prostate cancer cells suppresses tumorigenicity via induction of caspase-1-mediated apoptosis Cancer Res 1999 59 6 1366 1371 10096572

[B122] Winter RN Rhee JG Kyprianou N Caspase-1 enhances the apoptotic response of prostate cancer cells to ionizing radiation Anticancer Res 2004 24 3a 1377 1386 15274298

[B123] Hellawell GO Turner GD Davies DR Poulsom R Brewster SF Macaulay VM Expression of the type 1 insulin-like growth factor receptor is up-regulated in primary prostate cancer and commonly persists in metastatic disease Cancer Res 2002 62 2942 2950 12019176

[B124] Pollak M Insulin and insulin-like growth factor signalling in neoplasia Nat Rev Cancer 2008 8 915 928 10.1038/nrc2536 19029956

[B125] Rochester MA Riedemann J Hellawell GO Brewster SF Macaulay VM Silencing of the IGF1R gene enhances sensitivity to DNA-damaging agents in both PTEN wild-type and mutant human prostate cancer Cancer Gene Ther 2005 12 90 100 10.1038/sj.cgt.7700775 15499378

[B126] Benjamin W Turney Depletion of the type 1 IGF receptor delays repair of radiation-induced DNA double strand breaks Radiother Oncol 2012 103 402 409 10.1016/j.radonc.2012.03.009 22551565

[B127] Edwards DR Handsley MM Pennington CJ The ADAM metalloproteinases Mol Aspects Med 2008 29 5 258 289 10.1016/j.mam.2008.08.001 18762209 PMC7112278

[B128] Sung SY Kubo H Shigemura K Arnold RS Logani S Wang R Konaka H Nakagawa M Mousses S Amin M Anderson C Johnstone P Petros JA Marshall FF Zhau HE Chung LW Oxidative stress induces ADAM9 protein expression in human prostate cancer cells Cancer Res 2006 66 19 9519 9526 10.1158/0008-5472.CAN-05-4375 17018608

[B129] Peduto L Reuter VE Shaffer DR Scher HI Blobel CP Critical function for ADAM9 in mouse prostate cancer Cancer Res 2005 65 20 9312 9319 10.1158/0008-5472.CAN-05-1063 16230393

[B130] Fritzsche FR Jung M Tolle A Wild P Hartmann A Wassermann K Rabien A Lein M Dietel M Pilarsky C Calvano D Grutzmann R Jung K Kristiansen G ADAM9 expression is a significant and independent prognostic marker of PSA relapse in prostate cancer Eur Urol 2008 54 5 1097 1106 10.1016/j.eururo.2007.11.034 18061337

[B131] Josson S Anderson CS Sung SY Johnstone PA Kubo H Hsieh CL Arnold R Gururajan M Yates C Chung LW Inhibition of ADAM9 expression induces epithelial phenotypic alterations and sensitizes human prostate cancer cells to radiation and chemotherapy Prostate 2011 71 3 232 240 10.1002/pros.21237 20672324 PMC3174735

[B132] Inayat MS Chendil D Mohiuddin M Elford HL Gallicchio VS Ahmed MM Didox (a novel ribonucleotide Reductase inhibitor) overcomes Bcl-2 mediated radiation resistance in prostate cancer cell line PC-3 Cancer Biol Ther 2002 1 5 546 547 12496485 10.4161/cbt.1.5.174

[B133] McKenna WG Muschel RJ Gupta AK Hahn SM Bernhard EJ Farnesyltransferase Inhibitors as Radiation Sensitizers Semin Radiat Oncol 2002 12 3 27 32 10.1053/srao.2002.34866 12174342

[B134] Shi Y Wu J Mick R Cerniglia GJ Cohen-Jonathan E Rhim JS Koch CJ Bernhard EJ Farnesyltransferase inhibitor effects on prostate tumor micro-environment and radiation survival Prostate 2005 62 1 69 82 10.1002/pros.20122 15389805

[B135] Kelly WK O'Connor OA Krug LM Chiao JH Heaney M Curley T MacGregore-Cortelli B Tong W Secrist JP Schwartz L Richardson S Chu E Olgac S Marks PA Scher H Richon VM Phase I study of an oral histone deacetylase inhibitor, suberoylanilide hydroxamic acid, in patients with advanced cancer J Clin Oncol 2005 23 17 3923 3931 10.1200/JCO.2005.14.167 15897550 PMC1855284

[B136] Chinnaiyan P Vallabhaneni G Armstrong E Huang SM Harari PM Modulation of radiation response by histone deacetylase inhibition Int J Radiat Oncol Biol Phys 2005 62 1 223 229 10.1016/j.ijrobp.2004.12.088 15850925

[B137] Evers BM Neurotensin and growth of normal and neoplastic tissues Peptides 2006 27 2424 2433 10.1016/j.peptides.2006.01.028 16904238

[B138] Carraway RE Plona AM Involvement of neurotensin in cancer growth: evidence, mechanisms and development of diagnostic tools Peptides 2006 27 2445 2460 10.1016/j.peptides.2006.04.030 16887236

[B139] Amorino GP Deeble PD Parsons SJ Neurotensin stimulates mitogenesis of prostate cancer cells through a novel c-Src/Stat5b pathway Oncogene 2007 26 745 756 10.1038/sj.onc.1209814 16862179

[B140] Swift SL Burns JE Maitland NJ Altered expression of neurotensin receptors is associated with the differentiation state of prostate cancer Cancer Res 2010 70 347 356 10.1158/0008-5472.CAN-09-1252 20048080

[B141] Hassan S Dobner PR Carraway RE Involvement of MAP-kinase, PI3- kinase and EGF-receptor in the stimulatory effect of Neurotensin on DNA synthesis in PC3 cells Regul Pept 2004 120 155 166 10.1016/j.regpep.2004.03.004 15177934

[B142] Valerie NC Casarez EV Dasilva JO Dunlap-Brown ME Parsons SJ Amorino GP Dziegielewski J Inhibition of neurotensin receptor 1 selectively sensitizes prostate cancer to ionizing radiation Cancer Res 2011 71 21 6817 6826 10.1158/0008-5472.CAN-11-1646 21903767

[B143] Onozawa M Fukuda K Ohtani M Akaza H Sugimura T Wakabayashi K Effects of soybean isoflavones on cell growth and apoptosis of the human prostatic cancer cell line LNCaP Jpn J Clin Oncol 1998 28 360 363 10.1093/jjco/28.6.360 9730149

[B144] Hempstock J Kavanagh JP George NJ Growth inhibition of prostate cell lines in vitro by phyto-oestrogens Br J Urol 1998 82 560 563 10.1046/j.1464-410X.1998.00769.x 9806188

[B145] Kyle E Neckers L Takimoto C Curt G Bergan R Genistein-induced apoptosis of prostate cancer cells is preceded by a specific decrease in focal adhesion kinase activity Mol Pharmacol 1997 51 193 200 9203623 10.1124/mol.51.2.193

[B146] Hillman GG Wang Y Kucuk O Che M Doerge DR Yudelev M Joiner MC Marples B Forman JD Sarkar FH Genistein potentiates inhibition of tumor growth by radiation in a prostate cancer orthotopic model Mol Cancer Ther 2004 3 10 1271 1279 15486194

[B147] Yan SX Ejima Y Sasaki R Zheng SS Demizu Y Soejima T Sugimura K Combination of genistein with ionizing radiation on androgen-independent prostate cancer cells Asian J Androl 2004 6 4 285 290 15546018

[B148] Rashid A Liu C Sanli T Tsiani E Singh G Bristow RG Dayes I Lukka H Wright J Tsakiridis T Resveratrol enhances prostate cancer cell response to ionizing radiation. Modulation of the AMPK, Akt and mTOR pathways Radiat Oncol 2011 6 144 10.1186/1748-717X-6-144 22029423 PMC3217881

[B149] Xie D Gore C Liu J Role of DAB2IP in modulating epithelialto- mesenchymal transition and prostate cancer metastasis Proc Natl Acad Sci USA 2010 107 2485 2490 10.1073/pnas.0908133107 20080667 PMC2823864

[B150] Kong Z Xie D Boike T Raghavan P Burma S Chen DJ Habib AA Chakraborty A Hsieh JT Saha D Downregulation of human DAB2IP gene expression in prostate cancer cells results in resistance to ionizing radiation Cancer Res 2010 70 7 2829 2839 Epub 2010 Mar 23 10.1158/0008-5472.CAN-09-2919 20332235

[B151] Chen H Pong RC Wang Z Hsieh JT Differential regulation of the human gene DAB2IP in normal and malignant prostatic epithelia: cloning and characterization Genomics 2002 79 573 581 10.1006/geno.2002.6739 11944990

[B152] Igney FH Krammer PH Death and antideath: tumour resistance to apoptosis Nat Rev Cancer 2002 2 277 288 10.1038/nrc776 12001989

[B153] Oliver CL Miranda MB Shangary S Land S Wang S Johnson DE (−)-Gossypol acts directly on the mitochondria to overcome Bcl-2- and Bcl-X(L)-mediated apoptosis resistance Mol Cancer Ther 2005 4 1 23 31 15657350

[B154] Mohammad RM Wang S Aboukameel A Chen B Wu X Chen J Al-Katib A Preclinical studies of a nonpeptidic small-molecule inhibitor of Bcl-2 and Bcl-X(L) [(−)-gossypol] against diffuse large cell lymphoma Mol Cancer Ther 2005 4 1 13 21 10.1186/1476-4598-4-13 15657349

[B155] Xu L Yang D Wang S Tang W Liu M Davis M Chen J Rae JM Lawrence T Lippman ME (−)-Gossypol enhances response to radiation therapy and results in tumor regression of human prostate cancer Mol Cancer Ther 2005 4 2 197 205 10.4161/cbt.4.2.1441 15713891

[B156] Robinson TP Hubbard RB 4th Ehlers TJ Arbiser JL Goldsmith DJ Bowen JP Synthesis and biological evaluation of aromatic enones related to curcumin Bioorg Med Chem 2005 13 12 4007 4013 10.1016/j.bmc.2005.03.054 15911313

[B157] Notarbartolo M Poma P Perri D Dusonchet L Cervello M D'Alessandro N Antitumor effects of curcumin, alone or in combination with cisplatin or doxorubicin, on human hepatic cancer cells. Analysis of their possible relationship to changes in NF-kB activation levels and in IAP gene expression Cancer Lett 2005 224 1 53 65 10.1016/j.canlet.2004.10.051 15911101

[B158] Dorai T Gehani N Katz A Therapeutic potential of curcumin in human prostate cancer-I. Curcumin induces apoptosis in both androgen-dependent and androgen-independent prostate cancer cells Prostate Cancer Prostatic Dis 2000 3 2 84 93 10.1038/sj.pcan.4500399 12497104

[B159] Chendil D Ranga RS Meigooni D Sathishkumar S Ahmed MM Curcumin confers radiosensitizing effect in prostate cancer cell line PC-3 Oncogene 2004 26;23 8 1599 1607 10.1038/sj.onc.1207284 14985701

[B160] Yao D Natural IAP inhibitor Embelin enhances therapeutic efficacy of ionizing radiation in prostate cancer Am J Cancer Res 2011 1 2 128 143 21804946 PMC3144474

[B161] Jiang C Hu H Malewicz B Wang Z Lu J Selenite-induced p53 Ser-15 phosphorylation and caspase-mediated apoptosis in LNCaP human prostate cancer cells Mol Cancer Ther 2004 3 7 877 884 15252149

[B162] Husbeck B Peehl DM Knox SJ Redox modulation of human prostate carcinoma cells by Selenite increases radiation-induced cell killing Free Radic Biol Med 2005 38 1 50 57 10.1016/j.freeradbiomed.2004.09.022 15589371

[B163] Sun Y St Clair DK Xu Y Crooks PA St Clair WH A NADPH oxidase-dependent redox signaling pathway mediates the selective radiosensitization effect of parthenolide in prostate cancer cells Cancer Res 2010 70 2880 2890 10.1158/0008-5472.CAN-09-4572 20233868 PMC2848907

[B164] Milosevic M Warde P Ménard C Chung P Toi A Ishkanian A McLean M Pintilie M Sykes J Gospodarowicz M Catton C Hill RP Bristow R Tumor hypoxia predicts biochemical failure following radiotherapy for clinically localized prostate cancer Clin Cancer Res 2012 18 2108 2114 10.1158/1078-0432.CCR-11-2711 22465832

[B165] Vergis R Corbishley CM Norman AR Bartlett J Jhavar S Borre M Intrinsic markers of tumour hypoxia and angiogenesis in localized prostate cancer and outcome of radical treatment: a retrospective analysis of two randomised radiotherapy trials and one surgical cohort study Lancet Oncol 2008 9 342 351 10.1016/S1470-2045(08)70076-7 18343725

[B166] Joensuu G Joensuu T Nokisalmi P A phase I/II trial of gefitinib given concurrently with radiotherapy in patients with nonmetastatic prostate cancer Int J Radiat Oncol Biol Phys 2010 78 1 42 49 10.1016/j.ijrobp.2009.07.1731 20004525

[B167] Vuky J Pham HT Warren S Phase II study of long-term androgen suppression with bevacizumab and intensity-modulated radiation therapy (IMRT) in high-risk prostate cancer Int J Radiat Oncol Biol Phys 2012 82 4 e609 e615 Epub 2011 Dec 28 10.1016/j.ijrobp.2011.09.002 22208976

[B168] Ahmad IU Forman JD Sarkar FH Soy isoflavones in conjunction with radiation therapy in patients with prostate cancer Nutr Cancer 2010 62 7 996 1000 10.1080/01635581.2010.509839 20924975 PMC3856358

[B169] Kyprianou N Saacs JT Activation of programmed cell death in rat ventral prostate following castration Endocrinology 1988 22 552 562 2828003 10.1210/endo-122-2-552

[B170] Bolla M Van Tienhoven G Warde P Dubois JB Mirimanoff RO Storme G Bernier J Kuten A Sternberg C Billiet I Torecilla JL Pfeffer R Cutajar CL Van der Kwast T Collette L External irradiation with or without long-term androgen suppression for prostate cancer with high metastatic risk: 10-year results of an EORTC randomised study Lancet Oncol 2010 11 11 1066 1073 Epub 2010 Oct 7 10.1016/S1470-2045(10)70223-0 20933466

[B171] Hanks GE Pajak TF Porter A Grignon D Brereton H Venkatesan V Horwitz EM Lawton C Rosenthal SA Sandler HM Shipley WU Radiation Therapy Oncology Group Phase III trial of long-term adjuvant androgen deprivation after neoadjuvant hormonal cytoreduction and radiotherapy in locally advanced carcinoma of the prostate: the Radiation Therapy Oncology Group Protocol 92–02 J Clin Oncol 2003 21 3 972 978 10.1200/JCO.2003.11.023 14581419

